# Development and validation of the openness to the future scale: a prospective protective factor

**DOI:** 10.1186/s12955-018-0889-8

**Published:** 2018-04-23

**Authors:** C. Botella, G. Molinari, J. Fernández-Álvarez, V. Guillén, A. García-Palacios, R. M. Baños, J. M. Tomás

**Affiliations:** 1 Jaume I University, Av. Vicent Sos Baynat s/n, 12071 Castelló de la Plana, Spain; 2 CIBER of Physiopathology of Obesity and Nutrition, an initiative of ISCIII (CB06/03), Madrid, Spain; 3 Spanish Excellence Research Network PROMOSAM (PSI2014-56303-REDT), MINECO, Madrid, Spain; 4 University of Valencia, Av. Blasco Ibáñez, 21, 46010 Valencia, Spain; 5 Catholic University of the Sacred Heart, Milan, Italy

**Keywords:** Future-thinking, Assessment, Positive illusions, Prospective factors, Transdiagnostic, Optimism, Openness

## Abstract

**Background:**

Most of the research on psychopathology has provided an incomplete picture of mental health by focusing on vulnerability factors and omitting the transversal processes that may explain human adapted functioning. Moreover, research has not sufficiently addressed prospective protective factors for mental health. New theoretical and empirical endeavors aim to incorporate this perspective, particularly in the realm of emotional disorders. A positive view of the future is an indispensable process in attaining desired goals and wellbeing. Openness to the Future is a construct characterized by positive affectivity towards the future, which can be a protective factor for mental health. Although some scales assess future orientations, the complexity of this concept has not yet been captured; therefore, there is a need for new instruments. This study presents the development and validation of a scale for measuring Openness to the Future in clinical (*n* = 412) and community (*n* = 890) samples.

**Methods:**

Psychometric properties of the OFS were analyzed using Confirmatory Factor Analysis (CFA) and Item Response Theory (IRT) analyses, establishing cut-off points to better classify these two groups. Moreover, convergent and discriminant validity were examined by correlating the OFS with theoretically related constructs.

**Results:**

Results support a unidimensional structure and indicate that the items function similarly across clinical and community samples. Moreover, the Openness to the Future scale shows good convergent and discriminant validity.

**Conclusions:**

These findings suggest that the Openness to the Future scale is a valid and brief measure of openness to the future for use with clinical and community samples, and it could help to fill a gap in the literature regarding attitudes towards the future and their implications. Openness to the Future is presented as an empirically feasible and theoretically consistent construct that includes both prospective and protective factors in the psychopathological chart.

**Electronic supplementary material:**

The online version of this article (10.1186/s12955-018-0889-8) contains supplementary material, which is available to authorized users.

## Background

Most research on psychopathology has provided an incomplete picture of mental health by focusing mainly on vulnerability factors and omitting processes that may explain human adaptive functioning and well-being [1]. Moreover, protective prospective factors have not been sufficiently addressed. New theoretical and empirical endeavors aim to incorporate this comprehensive perspective, particularly in the realm of emotional disorders. A good example is the updated generic cognitive model [2], which postulates that psychological problems and clinical disorders are merely an accentuation of normal adaptive functioning. This model establishes a solid framework, not only to coherently conceptualize the dimensional nature of adaptability, but also to incorporate a set of principles, mechanisms, and formulations stemming from diverse theoretical roots, in order to better understand mental functioning [3]. The model comprehensively explains how information processing functions as a fundamental, core transversal process that leads to adaptive or maladaptive biases. From this perspective, the difference between normal and pathological functioning would be mainly quantitative. The interplay between information processing and the other transversal processes in the cognitive, affective, motivational, and behavioral systems may help to explain dysfunctional or positive functioning [2].

### Focus on vulnerability factors

The transdiagnostic perspective arose to examine the emergence and maintenance of psychopathological dysfunctions [4, 5], but this perspective has often neglected other processes that may be related to adaptation and normal functioning. Some classical psychological dimensions, such as extraversion, conscientiousness, or optimism [6–8], have been related to good mental health. However, the focus in psychopathology has been mainly on maladaptive dimensions, such as the construct of *anxious apprehension* proposed by Barlow [9, 10] as a fundamental element in anxiety disorders. The core psychopathology of this construct is a sense of uncontrollability of potentially threatening future events and the resulting state of helplessness. Thus, anxious apprehension is conceptualized as a *future-oriented* negative mood state in which an individual is cognitively, emotionally, and physiologically ready to deal with upcoming negative situations. The process comprises a shift in the individual’s attention to a self-evaluative focus, increased arousal, the activation of hypervigilance, and a set of cognitive biases. Barlow [9, 10] includes this sense of unpredictability and uncontrollability as the core of anxiety in the Triple Vulnerability Model.

This perspective agrees with the [2] *self-protective mode*, a network concerned with early detection and response to possible threats related to dangerous situations. In this case, the self-protective mode is a dimension that could comprise anxious apprehension. Another personality mode that is concerned with adaptation and the enhancement of personal resources is the *self-expansive mode* [2]. The negative pole of self-expansion is characterized by withdrawal, devaluation of the self, and conditional beliefs intimately linked to negative outcomes. These negative beliefs or pessimistic thoughts about the probability of obtaining expected or desired goals could lead to depression. The positive pole of the self-expansive mode is associated with the achievement of goals and objectives, increased self-esteem, and pleasure. The evaluation or devaluation of the self is a unifying theme in the self-expansive mode.

In sum, there are developments that propose transdiagnostic factors from a vulnerability perspective. However, the dimensional nature of mental health requires additional constructs that can conceptualize similar processes, but from a perspective of adaptation, normal functioning, and even flourishing [11]. Traditionally, psychology has mainly been interested in how people think, remember, interpret, and reconstruct the past; however, researchers are increasingly interested in how people think about the future [12]. Therefore, a shift in perspective to include a “forward-looking” framework that examines positive human functioning factors may be useful, and navigation into the future to develop more effective prospection (the mental representation of possible futures) is seen as a fundamental organizing principle [13–15].

### Focus on protective factors

Research has shown that normal individuals have three characteristics related to the future-oriented disposition: unrealistically positive views of themselves, an exaggerated belief in their ability to control their environment, and a view that their future will be far better than the average person’s [16, 17]. These three characteristics have been defined as *positive illusions*. Furthermore, individuals who are moderately depressed consistently show an absence of these illusions. Thus, the capacity to develop and maintain these positive illusions is considered an adaptive coping mechanism, especially in threatening circumstances, and it could be considered a valuable human resource that should be promoted, rather than an error-prone processing style that should be corrected [18].

People’s belief in their capacity to control things more than they really can is known as the *Illusion of Control* [16, 19], associated with important clinical outcomes such as less pain, less disability, and reduced depression [20]. By contrast, as in anxious apprehension, when the future is viewed as uncontrollable, threatening, or dangerous, the person can enter a state of helplessness [9, 10]. Therefore, clinical manifestations of anxiety can be interpreted as an extreme on a continuum, where clinically anxious individuals infer threats more readily than non-anxious individuals. However, from an evolutionary perspective, anxiety is an emotion that individuals need because it can counteract helplessness, forcing the individual to prepare to deal with possible future consequences [21]. In the same vein, sadness, the core feature of depressive states, should be considered an adaptive emotion for evolutionary purposes. However, when unjustified sadness appears recurrently, it may result in psychopathological forms due to faulty information processing interconnected with the affective, motivational, cognitive, and behavioral systems (e.g. [14, 22, 23])

From an evolutionary perspective, each person can have a different cognitive bias [24], which, depending on the interaction with contextual factors and the individual’s cognitive structure, may lead to adaptive or maladaptive functioning [2]. For example, estimates of future life events are often unrealistically optimistic: healthy individuals tend to overestimate the likelihood of positive events and underestimate the likelihood of negative ones [25, 26]. This phenomenon is known as the *optimism bias* and stems from the difference between a person’s expectations and the subsequent outcome. Data show that humans exhibit this optimism bias when anticipating what will happen to them in the future [27], and these optimistic illusions are the only kind of misbeliefs that are adaptive [28], or even crucial, from an evolutionary perspective [29]. The emergence of conscious foresight is thought to be indissolubly associated with the recognition of what awaits us as human beings in the future: aging, sickness, fading, and death. The logical fear (or terror) when faced with this perspective would be a “dead-end evolutionary barrier” that would interfere with all the daily activities necessary for survival. Therefore, the evolution of mankind might have ended without optimistic illusions [29], so that these illusions would be considered “normal” *evolved misbeliefs* [28].

Optimism is also related to a person’s belief in his/her control over future outcomes. Overestimating one’s control over events is thought to increase optimism, whereas not having a sense of control over the environment can induce depression [14, 30]. Individuals’ positive view of themselves and their future plays an important role in the way they engage with life and pursue and achieve goals [31]. Research has confirmed this enhanced positivity of future events and its relationship with personal goals and self-enhancement, regardless of time, distance, type of event, or age [32]. Carver, Scheier and Segerstrom proposed a self-regulatory model in which all human activity involves the identification and adoption of goals and the regulation of actions to achieve these goals [33, 34]. In addition, Liddell [21] emphasizes that the human capacity to make plans for the future and enjoy past achievements is the path to building culture [6].

### Openness to the future construct

In summary, the way we perceive and project our future has an influence on psychopathology, but also on health and well-being [14, 15, 17, 18, 20, 35]. Taking this into consideration, we propose a construct that implies showing openness and trust in the future. We define this construct as “Openness to the Future” (OF), an active cognitive-affective mood state that involves positive expectations about what life may bring, a sense of competence and ability to cope with events, the anticipation, planning and perseverance to reach an outcome even in the face of adversity, and the acceptance of what cannot be resolved or predicted. It means considering the future as a time that will arrive and is completely open, assuming multiple possibilities while being aware that what we do now (our actions, attitudes, plans, work, beliefs, etc.) greatly determines who we will be and what can happen to us later in life.

OF is conceptualized as a positive affective state with five domains: **(**1) *Illusion of Control*, a perceived sense of being in control and feeling confident about facing uncertain future situations; (2) *Acceptance****,*** being open and accepting what the future may bring; (3) *Engagement in life and planning*, the tendency to make plans and work to achieve them; (4) *Positive orientation toward the future,* the tendency to make positive interpretations about the future; (5) *Self-efficacy regarding future plans,* confidence in personal abilities to make plans and fulfil them. OF could be understood as “positive affectivity towards the future”. We propose that OF can protect individuals from the pathological process of emotional disorders. This argument agrees with proposals by Miloyan et al. [23] about the benefits of episodic foresight: the mental construction of threat-related future scenarios, even in the absence of present threat cues, enables one to plan, prepare, and more effectively manage the likelihood and/or consequences of a potential catastrophe. Moreover, it is compatible with the *pragmatic prospection* framework proposed by Baumeister, Vohs and Oettingen [13], which argues that *people think about the future in order to guide actions to bring about desirable outcomes*. This pragmatic prospection requires two steps: first, to imagine a desirable and optimistic outcome; and second, to anticipate steps, potential problems, and obstacles. Therefore, this second step involves a more cautious, and even pessimistic, approach, if the individual thinks that he/she cannot overcome obstacles along the way.

OF could lead to a flexible cognitive-affective structure in the individual, for example, by promoting the ability to flexibly upregulate or downregulate positive emotions based on one’s personal goals and the characteristics of the situation [36]. Thus, being open to the future could also generate new, more flexible, and adaptive patterns for solving problems. Following the broaden and build theory of positive emotions [37, 38], this positive affectivity towards the future could not only improve the experience of positive emotions in the present, but also help to strengthen the individual’s resources to cope with negative events and emotions in the future.

Although various measures of future-oriented thinking have been developed, most of them have focused on one specific component (e.g. cognitive future orientations [39, 40], optimism [41], hope [42] and hopelessness [43], intolerance to uncertainty [44], or positive illusions [45, 46]). Thus, the complexity of the OF construct has not yet been captured, and an integrative framework of these factors is needed. An essential aspect to take into consideration in developing such a complex measure is its brevity, both for research and practical purposes. For future research and development, particularly clinical research, brief but comprehensive future-oriented measures are important [47]. In fact, advances in prospective measures have been considered a priority for research [14].

For this reason, the goal of the current study is to develop the “Openness to the Future Scale” (OFS) and evaluate its psychometric properties in Spanish clinical and community samples, establishing cut-off points to better classify these two groups. Moreover, convergent and discriminant validity are examined by correlating the OFS with theoretically related constructs.

## Method

### Questionnaire development and item selection

A group of clinician-researchers with extensive experience working on psychological treatments designed to promote well-being (authors 1, 5 and 6) identified five potential relevant domains, considering previous questionnaires related to the research area (e.g. Life Orientation Test, Adult Hope Scale, General Self Efficacy Scale). Seven items were written in each domain, yielding thirty-five items in all, 15 positively worded and 20 negatively worded, reflecting the five relevant domains: (1) *Illusion of Control* (e.g., *“I think I have enough control over the direction my life takes”*); *(2) Acceptance* (e.g., *“I calmly accept that good and bad things will happen to me in life”*); (3) *Engagement in life and planning* (e.g., *“I have a lot of illusions and future plans”*); (4) *Positive orientation toward the future* (e.g., “*I feel hopeful about what the future may bring”*); and (5) *Self-efficacy towards the future,* (e.g.: *“I know that I will encounter obstacles in life, but I trust that I can overcome them”).* Items were randomly ordered to create the first version of the questionnaire. A five-point Likert-type scale was selected for item responses (from 1 “Strongly disagree” to 5 “Strongly agree”).^1^ Both the Spanish and English versions of the scale can be found in Additional files 1 and 2.

### Measures

#### Hopelessness

The Spanish version of the Beck Hopelessness Scale (BHS) [43, 48] consists of 20 true–false items that measure generalized negative expectations about the future. It can be used as an indicator of suicidal risk in depressed people. The BHS was selected as a good measure of discriminant validity. The alpha coefficient in the present study was .85.

#### Anxiety and depression

The Overall Anxiety Severity and Impairment Scale (OASIS) [49] was adapted for the Spanish-speaking population by the research group. It consists of 5 items that measure the frequency, severity, avoidance, and interference of anxiety on a 5-point scale. The alpha in the present study was .90.

Depression was measured with a Spanish adaptation of the Overall Depression Severity and Impairment Scale (ODSIS) [50]. The ODSIS consists of 5 items that measure the frequency, severity, avoidance, and interference of depression on a 5-point scale. The alpha in this study was .95. Anxiety and depression are both characterized by a tendency to generate and fixate on threat-related future scenarios, which would appear to be the opposite of OF.

*Worry.* The Spanish version of the Penn State Worry Questionnaire (PSWQ) [51] is a 16-item questionnaire that assesses self-reported trait worry on a Likert scale ranging from 1 (not at all typical of me) to 5 (very typical of me). The total score on the scale ranges from 16 to 80. It has shown good psychometric properties [52]. Worry is an apprehensive expectation about the future, characterized by negative emotions and thoughts about future outcomes. Therefore, the PSWQ was used as a measure of discriminant validity, and a negative correlation was hypothesized. In the present study, the internal consistency coefficient was .74.

#### Future expectations

The Subjective Probability Task (SPT) [53] was used as a measure of positive and negative future expectations. The SPT is divided into two subscales: 20 items refer to negative outcomes (e.g. “You will have a serious disagreement with a good friend”), and 10 items refer to positive outcomes (e.g. “You will make good and lasting friendships”) rated on a 7-point Likert scale from 1 (“not at all likely to occur”) to 7 (“extremely likely to occur”). An independent subtotal for each subscale has to be calculated. The negative expectancies subtotal score ranges from a minimum of 20 to a maximum of 140, whereas the positive expectancies subtotal score ranges from a minimum of 10 to a maximum of 70. The SPT has shown good internal consistency and discriminant validity. Our alphas were .93 for negative expectancies and .87 for positive expectancies.

#### Optimism

The *Life Orientation Test-revised* (LOT-R; [41]; Spanish version: [54]) assesses dispositional optimism and includes 10 items (4 of which are fillers) with a 5-point response scale (from 0 = strongly disagree to 4 = strongly agree). The total score ranges from 0 to 32. The alpha coefficient for the LOT-R in the present study was .72.

The LOT-R and the SPT were used to validate the OFS items examining a positive orientation towards the future. The LOT-R and the SPT-POS items would be positively correlated with the OFS, whereas the SPT-NEG items would be negatively correlated with it.

#### Positive and negative affect

The Positive and Negative Affect Schedule (PANAS) (Spanish version: [55]) consists of two subscales, with 10 items in each, that measure positive and negative affect. The PANAS subscales have been shown to be uncorrelated, with good internal consistency and test-retest reliability [56]. Positive affect items would be positively correlated with the OFS, whereas negative affect items would be negatively correlated with it. The alphas in this study were .90 for negative affect and .92 for positive affect.

#### Self-efficacy

The General Self Efficacy Scale-12 (GSES-12; [57]; Spanish version: [58]) has 3 factors: Initiative (willingness to initiate behavior), Effort (willingness to make an effort to complete the behavior), and Persistence (persevering to complete the task in the face of adversity). The total scale obtained a Cronbach’s α of .86.

#### Self-esteem

The Rosenberg Self-Esteem Scale (RSES) [59] is a unidimensional instrument to measure self-esteem that captures subjects’ global perception of their own worth on a 10-item scale, 5 positively worded items and 5 negatively worded items. The Spanish validation [60] was used in the present study. Only one item was used: “*On the whole, I am satisfied with myself”*. In the Spanish validation study, this item was able to discriminate as well as the total score.

The GSES-12 is a well validated measure of self-efficacy, and the RSES is one of the most widely used instruments to assess confidence in personal abilities. These measures were used to validate the OFS items examining confidence in personal abilities to make plans and fulfil them, and a positive correlation with the OFS was expected.

#### Psychological well-being

The Ryff Scales of Psychological Well-Being (RPWB) [61] focus on measuring multiple facets of psychological well-being, including autonomy, environmental mastery, personal growth, positive relations with others, purpose in life, and self-acceptance. Respondents rate statements on a scale from 1 to 6, with 1 indicating strong disagreement and 6 indicating strong agreement. For the present study, the Spanish adaptation of the 29-item version was used [62]. Internal consistency coefficients were .88 for autonomy, .73 for environmental mastery, .82 for personal growth, .85 for positive relations, .89 for purpose in life, and .88 for self-acceptance. Ryff’s Scale of Psychological Well-Being was used to cover different areas of eudaimonic well-being related to the OFS, and a positive correlation between the OFS and Ryff’s subscales was expected.

### Procedure

The community sample (CS) (university students and their relatives) was recruited from the university community in Spain (Valencian Community). In the case of university students, participants were recruited by posters on the University Campus and through the Internet and social media. Students completed the questionnaires in a designated room with a researcher present (authors 2 and 3). Twenty five groups of 30 students were assessed. Then, students were asked to inform their relatives about the research and encourage them to participate. Likewise, relatives of our research team members volunteered to participate.

Participants from the clinical population (CP) were attending two clinical services (Emotional Disorders Clinic at Universitat Jaume I and Previ Clinical Psychology Center) and had been diagnosed with emotional disorders (anxiety, depression) and/or personality disorders. They were receiving treatment when they were assessed. They were informed that a research study was being conducted, and they were invited to participate. Patients completed the questionnaires before treatment in a private office, and they were supervised by a researcher in case they needed any clarification.

Before the questionnaires were administered, demographic data were collected. Then, the OFS was administered, followed by the *Beck Hopelessness Scale (BHS)*, the *Life Orientation Test-Revised (LOT-R)*, the *General Self-efficacy Scale (GSES-12)*, the *Ryff Scales of Psychological Well-Being (RPWB)*, the *Rosenberg Self-Esteem Scale (RSES)*, the *Penn State Worry Questionnaire (PSWQ)*, the *Positive and Negative Affect Schedule (PANAS)*, the *Subjective Probability Task (SPT)*, the *Overall Anxiety Severity and Impairment Scale (OASIS)*, and the *Overall Depression Severity and Impairment Scale* (*ODSIS*). All measures were filled out by the participants, both in the clinical and non-clinical sample. Participants in the non-clinical sample took an average of 25 min to complete all the questionnaires. The average time for the clinical sample was 30 min. All participants provided voluntary and informed written consent. No specific inclusion and exclusion criteria were established, and no incentives were offered for participation. The research protocol was approved by the Ethical committee at the University Jaume I.

### Data analysis

Psychometric properties of the OFS were analyzed using two different statistical models, Confirmatory Factor Analysis (CFA), a procedure based on Classical Test Theory (CTT), and Item Response Theory (IRT) analyses. CFA models were estimated using the EQS program, version 6.1 with maximum likelihood and robust corrections (MLR), given the non-normality and five-point response scale. After establishing which model fit the observed data better, this model was tested separately in each group (CS and CP). After good fit had been determined for each group, a standard measurement invariance routine was applied by running a set of increasingly restrictive CFA and testing whether differences between these models were significant, either from a statistical or a practical point of view. In this case, a practical view was used to determine whether there were significant differences among the invariance models [63]. This set of models started with an unconstrained model tested simultaneously for both groups, the configural invariance model. The aim of this model was twofold: to test for configural equivalence (same structure), and to establish a baseline model fit to compare constrained models. Then, factor loadings were constrained across groups (weak or metric invariance). Metric invariance tests whether respondents in the two groups assign the same meaning to the factor under study. Finally, a model with constrained item intercepts is tested for strong or scalar invariance, which implies that both the meaning and the levels (intercepts) of the items are the same across groups, and, therefore, the level of the groups in the latent dimensions can be compared [64]. Model fit was evaluated using several criteria, specifically, the chi-square, CFI, TLI, and RMSEA. The following cut-offs were used to determine good fit: CFI and TLI above .90 (better if above .95) and RMSEA below .08 (better if below .05) [65].

In addition to these indexes, the acceptability of the model was evaluated according to the strength and interpretability of the parameter estimates and the absence of large and meaningful modification indices. Two theoretical structures were tested for the original 35 items on the OFS scale: a theoretical five-factor solution and a more parsimonious one-factor solution. However, model fit for the 35 items and one factor was still not adequate, and quite a large number of items had very poor psychometric properties (lack of consistency and discriminant power, for example). Given that the aim was to generate a manageable scale that would also have contents from the five theoretical dimensions in the construct, a first step consisted of item reduction. The 35 items were examined theoretically and psychometrically, in order to estimate how well the items fit the underlying construct. As mentioned above, the 35 items reflect five relevant content areas: Illusion of Control, Acceptance; Engagement in life and planning, Positive illusions, Positive orientation towards the future, and Self-efficacy. Accordingly, item reduction was conducted using both statistical (keeping the best behaving items using the criteria of factor loadings above .40, item-total correlations above .30, enough variability, and no floor or ceiling effects) and content-related criteria (to maintain the essential meaning of the five content areas). The initial CFA model for the original 35 items was crucial for item reduction. CTT models have been widely used in educational and psychological testing, but they have some limitations that can be overcome by IRT models. First of all, the two item statistics, which play essential roles in CTT models (difficulty and discrimination), are group dependent because they depend on the surveyed samples. Another important limitation of the CTT model is that the test score and true score are test dependent. In other words, the examinee’s score depends on a particular set of test items being administered. In IRT models, item parameters are group independent, and the values for each item parameter estimated from different groups of examinees are the same. Thus, the item parameters are group invariant. This property is important because if we have a part of the item curve, we can recover the rest of it. Another basic feature of IRT models is that the examinee’s ability is invariant with respect to the items used to determine it [66].

Graded Response Models [67], two-parameter IRT logistic models (2PL), were estimated with maximum likelihood and robust corrections. Mplus was also used to estimate the Graded Response Models. The Graded Response Model is an extension of the original 2PL model for use with polytomous items with no right answer [68]. 2PL models estimate two types of parameters for each item: discrimination (a) and difficulty (b). The discrimination parameter (a) determines the slope on which responses to the items change as a function of the “ability” level or latent construct measured. These slopes typically range from 0 to 3, and values above 1.0 are considered highly discriminant. Item difficulty (b) parameters determine how challenging each item is. Given that the OFS employs a 5-point rating scale, there are 4 response thresholds for each indicator. These thresholds indicate the level of the latent variable at which an individual has a 50% chance of scoring at or above a particular response category. Model fit of the Graded Response was established using the Likelihood Ratio Test (LRT) and several information criteria, specifically, the Akaike Information Criterion (AIC), the Bayesian Information Criterion (BCI), and the BCI adjusted version (ABIC).

The amount of measurement error was also estimated, using both CTT and IRT frameworks. In the case of CTT, the internal consistency of the OFS was estimated using the alpha and the composite reliability index (CRI), an index based on the confirmatory results that overcome some of the shortcomings of alpha as a good estimation of reliability [69]. Regarding the IRT framework, measurement accuracy was estimated with information functions: item and total information curves were calculated. These curves represent the amount of information an indicator or a scale provides across various levels of the latent variable. To assess classification accuracy, a Receiver Operating Characteristics curve (ROC curve) was used. The ROC curve is a plot that displays the trade-off between sensitivity (true positive rate) and specificity (false positive rate) on a series of cut-off points. The area under the ROC curve is considered an effective measure of the inherent validity of a diagnostic test. For our purposes, this curve is useful for (a) evaluating the test’s discriminatory ability to correctly distinguish between community sample participants and clinical participants; and (b) finding the optimal cut-off point to classify these groups. Additionally, t-tests and Chi square tests were calculated to compare the two subsamples. Moreover, correlations among the OFS and other variables of its nomological net were calculated. We hypothesized that the OFS would show a medium to high positive correlation with positive future expectancies, positive affect, self-efficacy, self-esteem, and psychological well-being; and a medium to high negative correlation with hopelessness and negative future expectancies, worry, anxiety, depression, and negative affect. These correlations were calculated in SPSS 21.

## Results

### Sample

The sample was composed of 1302 volunteers (75% women), 890 participants from the community (university students and their relatives) and 412 patients who had requested psychological treatment in a clinical center. Participants from the clinical population were individuals receiving treatment for emotional disorders (anxiety disorders and/or depression) (*n* = 192) or personality disorders (cluster b and c) (*n* = 220). The participants’ mean age was 27.49 years (SD = 10.11); 73.2% were single, 20.1% were married, and 6.8% were separated or divorced. With regard to their educational profile, 8.2% participants had elementary education, 22% had high school education, and 69.7% had a university degree. Regarding occupation, 53.4% were employed, 33.5% were unemployed, and 13% had never worked before.

### Group comparisons

Regarding the sociodemographic characteristics, significant differences were found in sex and age. The clinical group includes a larger proportion of women (88% vs 69%) (χ^2^(1) = 53.136, *p <* .001) than the community sample, and clinical patients were older than the community sample (31.6 years vs. 25.6 years old; (*t*(1252) = − 10.122, *p <* .001, d = − 0.82). Significant differences were also detected in educational level. The community sample had a higher educational level (76.6%) than the clinical group (53.7%) (χ^2^ (2) = 106.705. *p <* .001). No significant differences were detected in marital status. Regarding the other variables, as Table 1 shows, the community sample had lower BHS, OASIS, PANAS-N, and SPT-NEG scores, and higher scores on all the other measures used in the present study.

**Table 1:** Descriptive statistics of the OFS and all the study measures

	Clinical Population Mean (SD)	General Population Mean (SD)	*t*	*p*	*Cohen’s d [95% CI]*
OFS	32.29 (7.93)	39.02 (4.95)	15.85	<.001	−1.11 [− 1.23, −.99]
BHS	7.62 (5.91)	3.54 (3.22)	−9.64	<.001	.96 [.83, 1.08]
PSWQ	60.31 (12.11)	50.54 (13)	−7.86	<.001	.77 [.65, .89]
ODSIS	7.89 (5.37)	3.73 (4.13)	−8.32	<.001	.91 [.79, 1.03]
OASIS	9.66 (4.98)	5.02 (4.07)	−13.6	<.001	1.06 [.93, 1.18]
RSES	1.42 (0.93)	2.2 (0.72)	9.04	<.001	−.98 [−1.11, −.86]
GSES	27.07 (8.5)	33.17 (6.55)	9.88	<.001	−.84 [−.96, −.72]
LOT-R	16.02 (4.11)	20.65 (4.5)	7.05	<.001	−1.06 [−1.18, −.93]
PANAS-P	25.81 (8.82)	34.23 (7.24)	13.99	<.001	−1.08 [− 1.21, −.96]
PANAS-N	26.62 (8.61)	19.37 (6.85)	−12.41	<.001	.97 [.85, 1.10]
SPT-POS	41.79 (10.65)	50.18 (9.21)	9.7	<.001	−.86 [−.99, −.74]
SPT-NEG	77.53 (27.4)	67.53 (21.88)	−4.57	<.001	.42 [.30, .54]
BP-SA	5.18 (1.62)	5.8 (1.43)	4.41	<.001	−.41 [−.53, −.30]
BP-PR	4.19 (1.32)	4.87 (1.01)	5.65	<.001	−.61 [−.73, −.49]
BP-EM	3.61 (1.08)	4.34 (.87)	7.35	<.001	−.77 [−.89, −.65]
BP-A	3.67 (1.08)	4.4 (.94)	7.85	<.001	−.74 [−.86, −.62]
BP-PG	4.35 (1.28)	5.04 (.8)	5.96	<.001	.70 [−.82, −.59]
BP-PL	3.65 (1.32)	4.73 (.92)	9.08	<.001	−1.01 [−1.14, −.89]

Applying a Bonferroni correction *p* = .002 is needed to achieve a significant result. Accordingly, all *t*-tests were statistically significant. *OFS* Openness to the Future Scale; *BHS* Beck Hopelessness Scale. Score 0 to 3 (none or minimal); 4 to 8 (mild), *PSWQ* Penn State Worry Questionnaire. Total score ranges from 16 to 80; *ODSIS* Overall Depression Severity and Impairment Scale. Total score ranges from 0 to 20. Higher scores are indicative of greater depression-related severity and impairment, *OASIS* Overall Anxiety Severity and Impairment Scale. Total score ranges from 0 to 20. Higher scores are indicative of greater anxiety-related severity and impairment; *RSES* Rosenberg self-esteem scale. Item score ranges from 0 to 4. Higher scores indicate higher self-esteem; *GSES* General self-efficacy total scale. Total score ranges from 12 to 60. Higher scores indicate higher self-efficacy; *LOT-R* Life Orientation Test. Total score ranges from 0 to 32. Higher scores indicate higher dispositional optimism; PANAS-PA and PANAS-NA, positive and negative affect scale. Scores can range from 10 to 50 in both subscales. Higher scores representing higher levels of positive and negative affect; *SPT-POS and SPT-NEG* positive and negative future expectations. SPT-POS ranges from 10 to 70 and SPT-NEG from 20 to 140. Higher scores representing higher levels of positive and negative expectancies; *BP-A* Ryff Scales of Psychological Well Being, autonomy subscale (range 6–36); *BP-EM* environmental mastery (range 5–30), *BP-PG* personal growth (range 4–24), *BP-PR* positive relations with others (range 5–30), *BP-PL* purpose in life (range 5–30), *BP-SA* self-acceptance (range 4–24). For each subscale, a high score indicates that the respondent has a mastery of that area in his or her life

### Factor structure

First, two completely a priori CFA models were estimated for the 35 original items in the OFS. These models were: a) the theoretical a priori model with five dimensions, in which the dimensions were thought to be oblique (correlations among the dimensions were hypothesized and tested; b) and the second model, which was a more parsimonious, one-factor model that could also serve as a baseline model. Results clearly showed that the more parsimonious, one-factor model was a better representation of the data. Model fit for the five factor model was: χ^2^(550) = 8116.67, *p* < .001; RMSEA = .127, 90% CI [.124–.129]; CFI = .847; TLI = .838. This fit was not adequate, and moreover, the correlations among the factors were all large (*p* < .001). Some of them even cast doubt on the independence of the factors (three of them were larger than .8, one larger than .9, and, in all, 6 of the ten correlations were over .7). On the other hand, the one-factor model did not fit the data well either, but its fit was better than that of the one with five dimensions: χ^2^(560) = 5378.9, p < .001; RMSEA = .138, 90% CI [.136–.141]; CFI = .871; TLI = .858. Because the more parsimonious model fit the data from the 35 items better than the theoretical five-factor model, and the correlations among the dimensions were extremely large, the one-factor model was retained as the best representation of the items. However, given that model fit was still not adequate, and that a large number of items were not well related to this latent variable, after these confirmatory tests, item reduction was conducted. Levels of missing data (incomplete data) were under 7%, which may be considered a high response rate (Newman, 2009). This process led to a reduction in the initial 35 items to a total of 10 items that were core indicators of the construct, with two items per content area.

Model fit was considered with goodness-of-fit indexes, but also by carefully looking at the adequacy of parameter estimates according to theory and also a careful look at modification indexes, specifically values of more than 10 (*p* < .001). In the item reduction process, there were many modification indexes larger than 10. Most of them proposed cross-loadings. This information, along with the large correlations among the theoretical dimensions, made us attempt a drastic reduction of items with a unidimensional structure in mind. There are a number of reasons to retain two items per dimension. First, given that the results of 35-items pointed out that a one-factor solution better fit the data, there is no reason to have a long scale to measure an overall construct. Second, two indicators are a minimum in some statistical models (for example structural equation models) to identify a factor within a multivariate model. And third, two of the dimensions only had two items with adequate psychometric properties (factor loadings and reliability). Therefore, in order to maintain the balance of items per dimension, two items per dimension were chosen. These 10 items were the two best psychometrically behaving items for each of the five theoretical dimensions that tap openness to the future. These ten items were factor analyzed in both samples.

In the community sample, a single-factor model resulted in adequate model fit: χ^2^(35) = 137.08, *p* < .001; RMSEA = .058, 90% CI [.048–.068]; CFI = .979; TLI = .97. Factor loadings showed that all the items were strongly related to this factor, with values ranging from .43 to .79. We called this factor “Openness to the future”. In the clinical sample, the same single-factor model resulted in adequate model fit: χ^2^(35) = 172.56, p < .001; RMSEA = .100, 90% CI [.085–.115]; CFI = .966; TLI = .96. Each item displayed salient loadings on the latent factor (.34 to .82). The factorial invariance routine used the retained one-factor solution. Table 2 shows the sequence of models, their fit, and their differences from the baseline (configural) model. Given that the fit of the configural model was adequate, the sequence of nested models was then tested. Fit indices for the metric invariance model were similar to those of the configural model, with some showing even better fit (RMSEA) or negligible differences (ΔCFI< .01). Metric invariance was, therefore, tenable. Scalar invariance was then tested, and this model was better than the configural model, with a difference in CFI of .014.

**Table 2:** Set of hierarchical models to test for measurement invariance across general and clinical populations

Model	χ^2^	df	Δ χ^2^	Δdf	*p*	CFI	ΔCFI	RMSEA	90% CI	SRMR
Configural	274.42	70				.929		.068	.059–.076	.047
Metric	308.05	79	33.63	9	<.001	.921	.008	.067	.060–.075	.067
Scalar	373.73	87	99.31	17	<.001	.943	.014	.071	.063–.079	.073

*df* degrees of freedom, *Δ* differences

### Graded response model

The 2 parameter logistic model or graded response model was fitted to the data in both samples. Fit measures for the graded response model in the community group were: LRT (1997) = 544.24, *p* = 1, AIC = 3843.88, BIC = 3927.65, and ABIC = 3857.87. Model fit statistics and indices for the clinical group were: LRT (2007) = 625.42, p = 1; AIC = 3881.1, BIC = 3926.8; ABIC = 3888.8. Item discrimination and difficulty estimates are presented in Table 2. In both samples, all items had discrimination values above 1.0, and they can be considered highly discriminant, with the exception of item 6 (*“Sometimes I get scared and feel that I’m losing control when I think about what life may bring”*) in both samples and item 9 (*“I agree with the statement: every day is a new day”*) in the community sample. The most discriminant items were items 2 (*“I usually trust that things will work out”*) and 8 (*“I know I can overcome the obstacles I encounter in life”*) in both samples. The four difficulty parameters are also presented in Table 3. They are all monotonic, indicating adequate psychometric behavior.

**Table 3:** Means, standard deviations, item difficulty (a), and discrimination (b) of the 10 items in the OFS

	General Population						Clinical Population					
Item	M (SD)	a	b1	b2	b3	b4	M (SD)	a	b1	b2	b3	b4
1	3.98 (.84)	1.48	−6.24	− 3.31	− 1.89	1.46	3.18 (1.23)	1.72	−3.04	−1.07	.29	2.59
2	3.97 (.93)	2.16	−6.41	− 3.61	− 2.12	1.50	3.09 (1.26)	2.28	−3.22	−1.05	.55	3.05
3	3.89 (.91)	1.68	−5.66	−3.17	−1.67	1.69	2.76 (1.24)	2.08	−2.26	−.27	1.15	3.92
4	4.02 (.94)	1.47	−5.06	−3.35	−1.54	.91	3.10 (1.22)	1.66	−2.66	−1.07	.53	2.73
5	4.36 (.85)	1.32	−5.48	−3.74	−2.46	−.17	3.32 (1.38)	1.68	−2.50	−1.12	−.17	1.67
6	3.60 (1.26)	.83	−3.07	−1.32	−0.44	.87	2.94 (1.41)	.63	−1.52	−.19	.49	1.54
7	4.01 (.98)	1.04	−4.93	−2.57	−1.37	.71	3.21 (1.35)	1.59	−2.49	−1.05	.22	1.86
8	4.27 (.78)	2.46	−8.62	−5.70	−3.48	.57	3.42 (1.25)	2.78	−4.36	−2.01	−.34	2.61
9	4.11 (.99)	.92	−4.57	−2.63	−1.45	.35	3.83 (1.19)	1.32	−3.16	−2.26	−.91	.82
10	4.07 (.82)	1.36	−5.46	−3.93	−1.96	1.06	3.45 (1.18)	1.94	−3.43	−2.05	−.32	2.36

Means (M), standard deviations (SD)

**Fig. 1:**
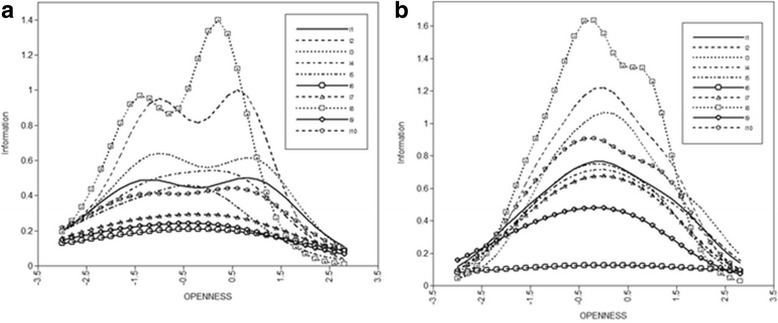
Item information Curves for all the items in the community group (**a**) and the clinical group (**b**)

**Fig. 2:**
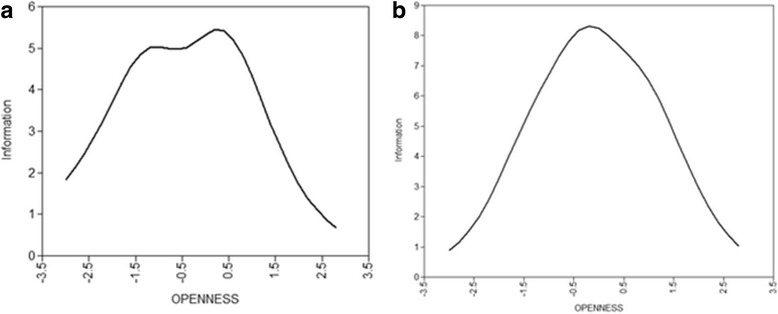
Item information curves for the community group (**a**) and the clinical group (**b**)

Item Characteristic Curves (ICC) for both samples are shown in Fig. 1. These ICCs, added up, form the test’s Total Information Curves (TIC), presented in Fig. 2 for both samples. Figure 2 reveals that the test gives more information about the clinical sample than it does about the community sample.

### Reliability: Internal consistency

Cronbach’s alphas for the 10-item scale were acceptable for both the clinical (.82) and community samples (.87). In addition to alphas, CRIs were also calculated, and results were .89 for clinical patients and .86 for the community group. According to the CRI, internal consistency seems adequate.

### ROC curve

**Fig. 3:**
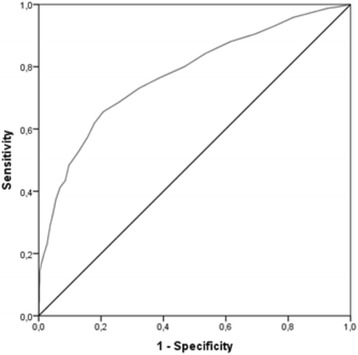
ROC curve

A ROC curve was generated, with the OFS total score as the continuous variable and the diagnostic status (clinical vs. community group) as the gold standard. Figure 3 shows the corresponding sensitivity and specificity (1-specificity) estimated in the ROC curve. A cut-off point from 37.5 to 38 on the OFS gave the best trade-off for sensitivity (.70) and specificity (.74). The area under the curve was .774 (SE = 0.80, *p* < .001, CI 95% of .746–.802). This area under the curve can be considered fair and almost good (values between .70 and .80 are considered fair, whereas values from .80 to .90 are considered good.

### Convergent validity

Table 4 summarizes the correlation coefficients. Positive correlations were found between the OFS and positive affect, positive future expectations, self-esteem, self-efficacy and psychological wellbeing. A negative correlation was found between the OFS and anxiety, depression, worry, negative affect, and negative future expectations. OFS was strongly associated with purpose in life and hopelessness.

**Table 4:** Correlation between the OFS and measures of depression, anxiety, worry, hopelessness, positive and negative affect, self-esteem, self-efficacy, future expectations, optimism, and psychological wellbeing

	ODSIS	OASIS	PSWQ	BHS	PA	NA	RSES	GSES	LOT-R	PE	NE	BP-A	BP EM	BP PG	BP PR	BP PL	BP SA
GP	−.23^**^	−.25^**^	−.27^**^	−.46^**^	.53^**^	−.37^**^	.33^**^	.50^**^	.54^**^	.44^**^	−.34^**^	.38^**^	.41^**^	.51^**^	.29^**^	.59^**^	.28^**^
CP	−.44^**^	−.41^**^	−.40^**^	−.74^**^	.58^**^	−.49^**^	.56^**^	.57^**^	.67^**^	.60^**^	−.48^**^	.41^**^	.63^**^	.67^**^	.43^**^	.76^**^	.33^**^

*GP* General population, *CP* Clinical population, *ODSIS* Overall Depression Severity and Impairment Scale, *OASIS* Overall Anxiety Severity and Impairment Scale, *PSWQ* Penn State Worry Questionnaire, *BHS* Beck Hopelessness Scale, *PA and NA* positive and negative affect scale, *RSES* Rosenberg self-esteem scale, *GSES* General self-efficacy total scale, *LOT-R* Life Orientation Test, *PE and NE* positive and negative future expectations, *BP-A* Ryff Scales of Psychological Well-Being, autonomy subscale, *BP-EM* environmental mastery subscale, *BP-PG* personal growth subscale, *BP-PR* positive relations with others subscale, *BP-PL* purpose in life subscale, *BP-SA* self-acceptance subscale

^**^ *p < .01*

## Discussion

The purpose of this study was to present openness to the future (OF), a construct characterized by positive affectivity towards the future that can be a prospective protective factor for mental health, and the development and validation of the Openness to the future scale (OFS), designed to operationalize and measure this construct.

The OF construct is theoretically considered a protective factor and, thus, should discriminate between clinical and general populations. At the core of this construct is the capacity to perceive the future and life simply as “life”, viewing it as something “normal” (rather than threatening), a time that will arrive, and completely open. People who are open to the future perceive themselves as having the ability to live life with confidence (which implies being able to accept anything life brings) and the capacity to feel illusion and continue to feel optimistic about life and events and plan steps to achieve objectives. These people have the ability to engage in daily meaningful activities and plans, perceiving challenges as opportunities rather than threats. This construct would be related to the *self-expansive mode* presented by Beck and Haigh [2, 43]. Therefore, OF may help to broaden the repertoire of an individual’s thoughts, capacities, and actions, and it may also lead to decreasing the constraints or limitations (e.g. avoidance, escape) that negative emotions can cause, as in the negative relationship between helplessness and OF, particularly in the clinical group. The OF construct is consistent with expectancy-value theories [70, 71] that encompass two premises: (a) the behavior reflects the pursuit of goals, and (b) the confidence that a goal can be achieved reflects a positive expectancy about future outcomes. OF tries to expand these models. This construct would also be related to the Miloyan et al. [23] proposal about the benefits of episodic foresight, and to the pragmatic prospection framework proposed by Baumesteir et al. [13].

The operationalization of this construct can be a good measure of protective human resources and a positive indicator of mental health and psychological adjustment [72] or, on the contrary, an indicator of psychopathology such as depression and anxiety. This paper describes the development and validation of the OFS, a measure designed to assess OF. In order to study the psychometric properties of this instrument, Classical Test Theory (CTT) and Item Response Theory (IRT) approaches were combined. IRT has the advantage that it allows for more realistic assumptions about measurement error, and it offers parameter estimates that are sample independent [73]. However, it has some shortcomings. For example, Zickar and Broadfoor [74] mentioned its need for large sample sizes and its strong assumption about unidimensionality. They also demonstrated that the CTT approach has tools such as structural models, which are not available in IRT models and justify the application of IRT models. The CFA (structural model framework) gives important information about unidimensionality and equal discrimination parameters. In other words, psychometric results from CTT and IRT frameworks reinforce each other.

With regard to the factor structure and dimensionality of the OFS, the original item bank of 35 items, 7 from each of the 5 relevant content areas (Illusion of Control, Acceptance, Engagement in life, Positive orientation, and Self-efficacy) was first reduced to the 10 core indicators of the OF construct, including two items per content dimension. Results from CFA suggested a good fit for a unidimensional factor structure in both clinical and community samples. Further tests of measurement invariance in both samples were then performed. This invariance routine showed that the one-factor structure of the OFS was not only metric invariant, but also scalar invariant, in both samples. This is important given that meaningful group comparisons are only tenable when scalar invariance is present among the groups. Within the CTT framework, internal consistency was also estimated, and the OFS was shown to be a reliable measure in clinical and general population samples, at both the scale (alphas) and item levels (factor loadings).

With regard to the graded response model (IRT), results showed that the OFS has highly discriminant items, items 2 (*“I usually trust that things will work out”*) and 8 (*“I know I can overcome the obstacles I encounter in life”*) in both samples, and that the difficulty parameter was adequate. However, the IRT model also indicated that the amount of information given by the test was greater for the clinical sample than for the community group. Nevertheless, ROC analysis allowed us to estimate the cut-off point that best discriminates (and predicts) between individuals with and without psychological disorder diagnoses (clinical and community samples). A cut-off point of 37.5 on the OFS correctly achieved the necessary sensitivity/specificity criterion.

Regarding convergent and discriminant validity, OFS was positively related to a variety of positive measures (positive affect, self-esteem, self-efficacy, optimism, positive future expectancies, and psychological well-being), and negatively associated with measures of anxiety, depression, worry, negative future expectancies, and negative affect, in both clinical and community samples. Moreover, the OFS also demonstrated a large negative correlation with a well-validated indicator of hopelessness (BHS) and a large positive correlation with one of the dimensions of psychological well-being, the belief that one’s life is purposeful and meaningful (BP-PL) [58]. These associations were higher in the clinical sample than in the community sample. From a theoretical point of view, hopelessness, purpose in life, and OF have several commonalities given their shared focus (specifically, their future orientation). In this sense, we may expect that individuals high in OF would have greater confidence in their ability to attain goals and a sense of directedness, therefore, this contributes to the belief of a purposeful life. On the contrary, individuals low in OF would have difficulties in making future and significant plans, with a tendency to make more negative interpretations about what the future may bring. These results encourage us to continue analyzing the differential effects of the future thinking constructs and that was the purpose of the development of the OFS. Future research has to investigate how hopelessness, purpose in life and OF relate to each other. Our findings contribute to a large and growing body of literature indicating that people who have higher scores on measures of positive future thinking show less psychopathology and better emotional and subjective well-being [14, 33], and they engage in more purposeful and meaningful activity. OF may be an important variable to examine further, in order to better understand psychological functioning and well-being [75].

OF can be considered a transdiagnostic process due to its dimensional nature from adaptability to maladaptation. However, this has to be confirmed by additional empirical research. The model presented by Beck and Haigh [2] highlights the continuity between adaptation and maladaptation, and it provides an articulation to better conceptualize and test this dimensionality through the introduction of the theory of modes. Based on this model, it is possible to include protective factors that have traditionally been underestimated, such as OF.

One contribution of this study is related to the current discussion about transdiagnostic approaches, both in terms of psychopathological understanding and clinical implications. As Harvey et al. [76] stated, at least two distinctions can be made in transdiagnostic approaches: processes in which causal effects can be found, including how they work (mechanistic processes); and processes that are identified in diverse clinical profiles, but lack these additional explanations (descriptive processes). Taking our data into account, OF could be considered a “descriptively transdiagnostic” construct, present in diverse clinical samples (Botella et al. manuscript under preparation). As a future line of research, it would be relevant to study whether the OF construct could be considered a “mechanistically transdiagnostic” construct to be taken into consideration as a shared mechanism in the treatment of emotional disorders [5], providing the basis for the construction of prospection-based techniques [14].

An additional aspect that remains to be studied consists of determining the possible conceptualization of OF as a change mechanism in the treatment of emotional disorders. As Lemmens, Müller, Arntz and Huibers [77] stated, 39 potential mediators in 12 different treatment modalities have been studied for depression, but apart from hopelessness, none of them have considered the prospective affective, cognitive, and motivational disposition. With regard to anxiety disorders, a greater emphasis is placed on the prospective dimension due to the core emotion involved, which is precisely future-oriented (i.e. intolerance to uncertainty) [78]. Even so, the number of studies that explicitly refer to intolerance to uncertainty is low. Thus, including future-oriented variables could help to identify mediators that, in turn, would be useful in establishing shared treatment mechanisms. An important step in this direction is the recognition that prospection is a crucial and under-appreciated transdiagnostic process in the study of depression, and possibly in other psychological disorders and chronic diseases such as chronic pain. Therefore, it is necessary to determine whether and how the patient’s faulty prospection drives or shapes psychopathology, and how pragmatic prospection can be improved [13, 14].

This study has strengths and limitations. One strength is the presentation of OF, a new future-oriented construct that can be useful from both research and applied points of view. A second strength is the development of a brief scale to operationalize and measure this prospective construct, validated not only in community samples, but also with clinical groups that suffer from different mental disorders. Finally, another strength is the use of several statistical frameworks to study the scale’s psychometric properties, IRT, CTT, and Roc analysis, and the large sample sizes included. Among the limitations, first, the development of the potential domains of the OFS and the items related to each was carried out only by the authors of the study. Nevertheless, this process was conducted based on a thorough search of the scientific literature and using a method similar to that of other studies designed to develop and validate similar instruments [79, 80]. Second, the OFS was not administered repeatedly over time in the present study. Thus, evaluations of test–retest reliability or sensitivity to change were not possible. Considering the large number of additional instruments to measure convergent and divergent validity, a further limitation is the lack of a pilot study that could have provided preliminary results about the acceptability of the study with the full battery of instruments. Furthermore, it would have been ideal to perform cognitive interviews to assess the acceptability and comprehension of the items’ wording and instructions. However, experienced clinicians and researchers supervised the completion of the scale, and none of the participants expressed difficulties in understanding any of the items. Another limitation is that the community sample was limited to university students and their families, without excluding them if they had any psychological problems. We acknowledge that this was not an optimal sample recruitment procedure, but our aim was to obtain a more representative sample of the community group. Finally, another limitation is that the scale has only been validated in Spanish. Future studies will have to validate it in samples that speak other languages. Due to its ability to distinguish between clinical and community groups, future studies should analyze whether a person’s level of OF is a stable trait, or whether it is susceptible to life events, circumstances, or psychological intervention. In this sense, it would be interesting to test change sensitivity and measurement invariance of the OFS over time.

All emotions, cognitions, emotion regulation strategies, and any other psychological processes, including the new OF construct, tend to be adaptive as long as the individual has the flexibility to explicitly deploy the necessary resources [81, 82]. For example, anxiety and sadness are considered relevant emotions that can be adaptive or maladaptive [2]. OF must also be contemplated from this dimensional perspective, taking into account the possible detrimental effects of extreme levels of this construct and whether they could potentially exacerbate or cause psychopathology.

As McGinn and Hofmann stated [3], Cognitive Behavior Therapy (CBT) is effective, but it also has many shortcomings and challenges. Thus, it is necessary to deepen the connection between psychopathological advances and clinical applications by incorporating new developments that can stem from different theoretical perspectives. OF is constructed with this in mind, and its core essence undoubtedly lies in cognitive theory, not only adding traditional behavioral integrated contents (self-efficacy), but also humanistic principles (much more related to self-engagement and acceptance). Furthermore, this construct broadens the traditional focus on the dysfunctional realm because it is conceptualized as a protective factor from the prospective dimension, a crucial area of study in the coming years.

This construct is not only relevant for understanding normal and psychopathological functioning, but it also represents an essential step in incorporating prospective aspects into therapeutic approaches. In terms of the way the OFS could be used in clinical practice and research, the operationalization and assessment of this construct may provide a useful measure of human resources and a positive indicator of mental health and psychological adjustment. If the OFS is shown to express a protective factor in different psychological disorders, it could be very useful for making decisions in case formulation, treatment prognosis, and the treatment plan. For example, if appropriately supported with further empirical evidence, low levels of OF could indicate the need to incorporate more therapeutic contents related to positive affect and positive emotions, in order to teach people how to improve those aspects that do not develop naturally. The development of prospective-based techniques may enhance current transdiagnostic treatment strategies for depression [14] and other disorders. However, this can only be achieved after rigorous theoretical and empirical understanding of the constructs involved, as in the case of OF. Moving forward, the construct and the measure might be useful in relation to several problems (e.g. sleep, pain, substance use etc.), and age, work status, education, and health literacy may be important variables in individuals’ level of OF. As this study shows, this important research program is already under way [83].

## Conclusions

In sum, OF is a new and promising psychological construct that can be useful to improve our knowledge about psychopathology and normal human functioning, and it can be related to other relevant constructs such as purpose in life, spirituality, and meaningful daily activity. In addition, OF re-conceptualizes positive future expectations in a way that incorporates a positive illusion of control, an active process of acceptance of future scenarios, and confidence and engagement in the personal capacity to make plans to obtain desired results and cope with adversity. The OFS is a promising brief instrument to measure this positive affective orientation towards the future, and it shows adequate factorial validity, scalar invariance, reliability, and convergent and discriminant validity in clinical and general samples. This measure now has to be tested in specific clinical populations to determine whether or not the OFS can assist in the management and treatment of patients, and which personal characteristics (such as health and employment status, relationships, etc.) are affecting this measure.

## Additional files

Additional file 1: Openness to the future Scale. (DOCX 22 kb)

Additional file 2: Escala de Apertura hacia el Futuro. (DOCX 16 kb)

## Footnotes

1 Although the instrument was developed in Spanish and the entire sample consisted of native Spanish speakers, we performed translation and back-translation for the current study in order to ensure that the OFS can potentially be used and validated in other samples. The person involved in the translation process is a native English speaker who has been living in Spain for many years and is fluent in both languages. In addition, the whole process was supervised by researchers with experience in the field (authors 1, 5 and 6), who are also the original developers of the instrument. Both the Spanish and English versions of the scale can be found in Additional files 1 and 2.
