# Supplementary material for: Development and validation of the openness to the future scale: a prospective protective factor
Source: Health Qual Life Outcomes. 2018 Apr 23;16:72. doi: 10.1186/s12955-018-0889-8 (PMC5914035; doi:10.1186/s12955-018-0889-8)
Supplement: Supplementary file 1 — Openness to the future Scale. (DOCX 22 kb) [file 12955_2018_889_MOESM1_ESM.docx]

**Additional file 1:** Openness to the future Scale

| Below, you will find different statements that you can identify with to a greater or lesser degree. Please, indicate your level of agreement or disagreement with each of them, based on the following scale: |
| --- |

| 1  Strongly disagree | 2  Somewhat disagree | 3  Neither agree nor disagree | 4  Somewhat agree | 5  Strongly agree |
| --- | --- | --- | --- | --- |

**There are no right or wrong answers. The important thing is what you think. Do not spend a long time thinking about the exact meaning of the statements. Just give the first answer that best fits your way of thinking.**

| 1. When I make plans, I am sure I will be able to carry them out. | 1 | 2 | 3 | 4 | 5 |
| --- | --- | --- | --- | --- | --- |
| 2. I usually trust that things will work out | 1 | 2 | 3 | 4 | 5 |
| 3. I think I have enough control over the direction my life takes. | 1 | 2 | 3 | 4 | 5 |
| 4. I am very excited about future opportunities and challenges | 1 | 2 | 3 | 4 | 5 |
| 5. I have a lot of illusions and future plans. | 1 | 2 | 3 | 4 | 5 |
| 6. Sometimes I get scared and feel that I’m losing control when I think about what life may bring | 1 | 2 | 3 | 4 | 5 |
| 7.  I calmly accept that good and bad things will happen to me in life | 1 | 2 | 3 | 4 | 5 |
| 8. I know I can overcome the obstacles I encounter in life. | 1 | 2 | 3 | 4 | 5 |
| 9. For me: every day is a new day. | 1 | 2 | 3 | 4 | 5 |
| 10. I feel hopeful about what the future may bring. | 1 | 2 | 3 | 4 | 5 |

Correction key: A total Openness to the Future score is obtained by adding all items scores; Item 6 is formulated inversely and thus the score must be inverted (1 = 5, 2 = 4, 3 = 3, 4= 2 and 5 = 1). Permission of Botella et al. (2017).
