# Supplementary material for: Development and validation of the openness to the future scale: a prospective protective factor
Source: Health Qual Life Outcomes. 2018 Apr 23;16:72. doi: 10.1186/s12955-018-0889-8 (PMC5914035; doi:10.1186/s12955-018-0889-8)
Supplement: Supplementary file 2 — Escala de Apertura hacia el Futuro. (DOCX 16 kb) [file 12955_2018_889_MOESM2_ESM.docx]

**Additional file 2:** Escala de Apertura hacia el Futuro

| A continuación, encontrarás diferentes frases con las que te puedes sentir identificado en mayor o menor medida. Por favor, indica el grado de acuerdo o de desacuerdo que tienes con cada una de ellas según la siguiente escala: |
| --- |

| 1  Totalmente en desacuerdo | 2  Algo en desacuerdo | 3  Ni de acuerdo, ni en desacuerdo | 4  Algo de acuerdo | 5  Totalmente de  acuerdo |
| --- | --- | --- | --- | --- |

**No hay respuestas correctas ni incorrectas. Lo importante es lo que tú opines.
No te detengas mucho a pensar en el significado exacto de las afirmaciones, contesta lo primero que creas que se ajusta más a tu forma de pensar.**

| 1. Cuando hago planes, estoy seguro de que conseguiré llevarlos a cabo. | 1 | 2 | 3 | 4 | 5 |
| --- | --- | --- | --- | --- | --- |
| 2. Suelo confiar en que las cosas saldrán bien. | 1 | 2 | 3 | 4 | 5 |
| 3. Creo que tengo bastante control sobre el rumbo que toma mi vida. | 1 | 2 | 3 | 4 | 5 |
| 4. Los desafíos y los retos hacia el futuro me resultan muy estimulantes. | 1 | 2 | 3 | 4 | 5 |
| 5. Tengo un montón de planes y de ilusiones. | 1 | 2 | 3 | 4 | 5 |
| 6. A veces me asusto y siento que pierdo el control cuando pienso en lo que podrá depararme la vida. | 1 | 2 | 3 | 4 | 5 |
| 7. Acepto con tranquilidad que en la vida me van a ocurrir cosas buenas y malas. | 1 | 2 | 3 | 4 | 5 |
| 8. Sé que la vida me presentará obstáculos, pero confío en que podré superarlos. | 1 | 2 | 3 | 4 | 5 |
| 9. Estoy de acuerdo con la afirmación: cada día es un nuevo día. | 1 | 2 | 3 | 4 | 5 |
| 10. Tengo esperanza por lo que pueda traer el futuro. | 1 | 2 | 3 | 4 | 5 |

*Clave de corrección:* Se obtiene una puntuación total de Apertura hacia el Futuro sumando las puntuaciones de todos los items; el items 6 está formulado de forma inversa y por tanto debe invertise la puntuación (1 = 5, 2 = 4, 3 = 3, 4 = 2 y 5 = 1). Reproducido con permiso de Botella et al. (2017).
